# Influence of number of membership functions on prediction of membrane systems using adaptive network based fuzzy inference system (ANFIS)

**DOI:** 10.1038/s41598-020-73175-0

**Published:** 2020-09-30

**Authors:** Meisam Babanezhad, Armin Masoumian, Ali Taghvaie Nakhjiri, Azam Marjani, Saeed Shirazian

**Affiliations:** 1 Institute of Research and Development, Duy Tan University, Da Nang, 550000 Vietnam; 2 Faculty of Electrical–Electronic Engineering, Duy Tan University, Da Nang, 550000 Vietnam; 3 Departament d’Enginyeria Informàtica i Matemàtiques, Universitat Rovira i Virgili, Avda. Paisos Catalans, 26, 43007 Tarragona, Spain; 4 Department of Petroleum and Chemical Engineering, Science and Research Branch, Islamic Azad University, Tehran, Iran; 5 Department for Management of Science and Technology Development, Ton Duc Thang University, Ho Chi Minh City, Viet Nam; 6 Faculty of Applied Sciences, Ton Duc Thang University, Ho Chi Minh City, Viet Nam; 7 Department of Chemical Sciences, Bernal Institute, University of Limerick, Limerick, Ireland; 8 Laboratory of Computational Modeling of Drugs, South Ural State University, 76 Lenin prospekt, 454080 Chelyabinsk, Russia

**Keywords:** Chemical engineering, Mathematics and computing

## Abstract

In membrane separation technologies, membrane modules are used to separate chemical components. In membrane technology, understanding the behavior of fluids inside membrane module is challenging, and numerical methods are possible by using computational fluid dynamics (CFD). On the other hand, the optimization of membrane technology via CFD needs time and computational costs. Artificial Intelligence (AI) and CFD together can model a chemical process, including membrane technology and phase separation. This process can learn the process by learning the neural networks, and point by point learning of CFD mesh elements (computing nodes), and the fuzzy logic system can predict this process. In the current study, the adaptive neuro-fuzzy inference system (ANFIS) model and different parameters of ANFIS for learning a process based on membrane technology was used. The purpose behind using this model is to see how different tuning parameters of the ANFIS model can be used for increasing the exactness of the AI model and prediction of the membrane technology. These parameters were changed in this study, and the accuracy of the prediction was investigated. The results indicated that with low number of inputs, poor regression was obtained, less than 0.32 (R-value), but by increasing the number of inputs, the AI algorithm led to an increase in the prediction capability of the model. When the number of inputs increased to 4, the R-value was increased to 0.99, showing the high accuracy of model as well as its high capability in prediction of membrane process. The AI results were in good agreement with the CFD results. AI results were achieved in a limited time and with low computational costs. In terms of the categorization of CFD data-set, the AI framework plays a critical role in storing data in short memory, and the recovery mechanism can be very easy for users. Furthermore, the results were compared with Particle Swarm Optimization (PSOFIS), and Genetic Algorithm (GAFIS). The time for prediction and learning were compared to study the capability of the methods in prediction and their accuracy.

## Introduction

The separation processes developed based on membrane technology causes the creation of a new method to separate chemical components, and the method is completely different from conventional separation techniques existed in chemical/biochemical industries, especially in the purification. This method of purification can separate different components, and it can be used in small scales comparing to conventional technologies. The separation process in membrane technology results in several advantages compared to other conventional methods and purification systems, such as low separation cost, modular design, and low energy demand^1,2^.

This technology can be used in small channels (micro scale) that is an example of small scales or microscopic observation, but the technology creates a high capacity in the separation of different components in membrane technology. Among all membrane systems, the membrane contactor systems can separate chemical components and create reactions among chemical components in a very small domain. Other applications of membrane contactors incude: membrane crystallization^3,4^, wastewater treatment^5,6^, liquid extraction^7,8^, and gas absorption^9–11^.

Designing a membrane technology is possible in different mathematical and numerical methods such as computational fluid dynamics (CFD), mathematical and physical approximations, such as mechanistic modeling^12–14^. Recently, numerical methods or CFD were used as a tool for modeling the internal layers of the membrane system, and therefore, the mass transfer and chemical reaction among chemical components and membrane can be fully understood. CFD could model for the fluid flow and interaction among different phases, including solid, gas, and liquid. The CFD tools also could measure the heat and mass transfer in a system. CFD has widespread applications in different industries, including the industries that use membrane technology such as wastewater treatment. As far as the fluid movement and the interaction between the phases are two significant parameters in the membrane technology, the CFD could be a suitable tool for a better understanding of the process. CFD also enables the researchers to model and optimize different components of the membrane system; for instance, the geometry size of the membrane system can be optimized by using CFD simulations. Also, CFD enables us to do the numerical method for solving from small to large scales. CFD could provide us with exact understanding of membrane technology by the complex solving of the Navier–Stokes equations, and coupling the Navier–Stokes and mass transfer equation leading to a better understanding of the separation of components in a process. Artificial intelligence (AI) and CFD solution are used together due to the high costs of using CFD for optimizing the membrane technology and long time for designing a membrane technology via CFD, boundary limitation and complex flow conditions of CFD, and numerical instability; therefore, AI methods are trained from CFD and after the training process, AI provides the results in the different domains^15,16^. By creating the CFD results, AI can provide us with another solution that is faster than CFD. The solution is also far from numerical instability, complex boundary conditions that they existed in CFD. This stability in the calculation of AI is tightly coupled with “non-sense learning” mechanism in the AI method which means, AI can only train the dataset, and it cannot understand the physics behind the process, including the complex boundary conditions^17^. Recently, An adaptive neuro-fuzzy inference system (ANFIS) was used, which is a combination of fuzzy logic system and neural networks^18^. The method enables the researchers to transfer numerical results of CFD to the AI domain, and the local points in the membrane system could be predicted via AI.

Owing to this point that AI needs sensitivity to reach an exact and reliable solution that can be used many times in optimization of the membrane technology, training of the AI could be done in different ways, and changing the tuning parameters is needed in training that are the functions, or the number of nodes^19^. To do so, in the current research, the researchers study AI and change parameters in AI, including the number of inputs in training, membership functions to achieve an exact prediction that is reliable for the optimization process. The results were compared with Particle Swarm Optimization (PSOFIS), and Genetic Algorithm (GAFIS). The time for prediction and learning were compared to study the capability of the methods in prediction and their accuracy.

## CFD method

**Figure 1:**
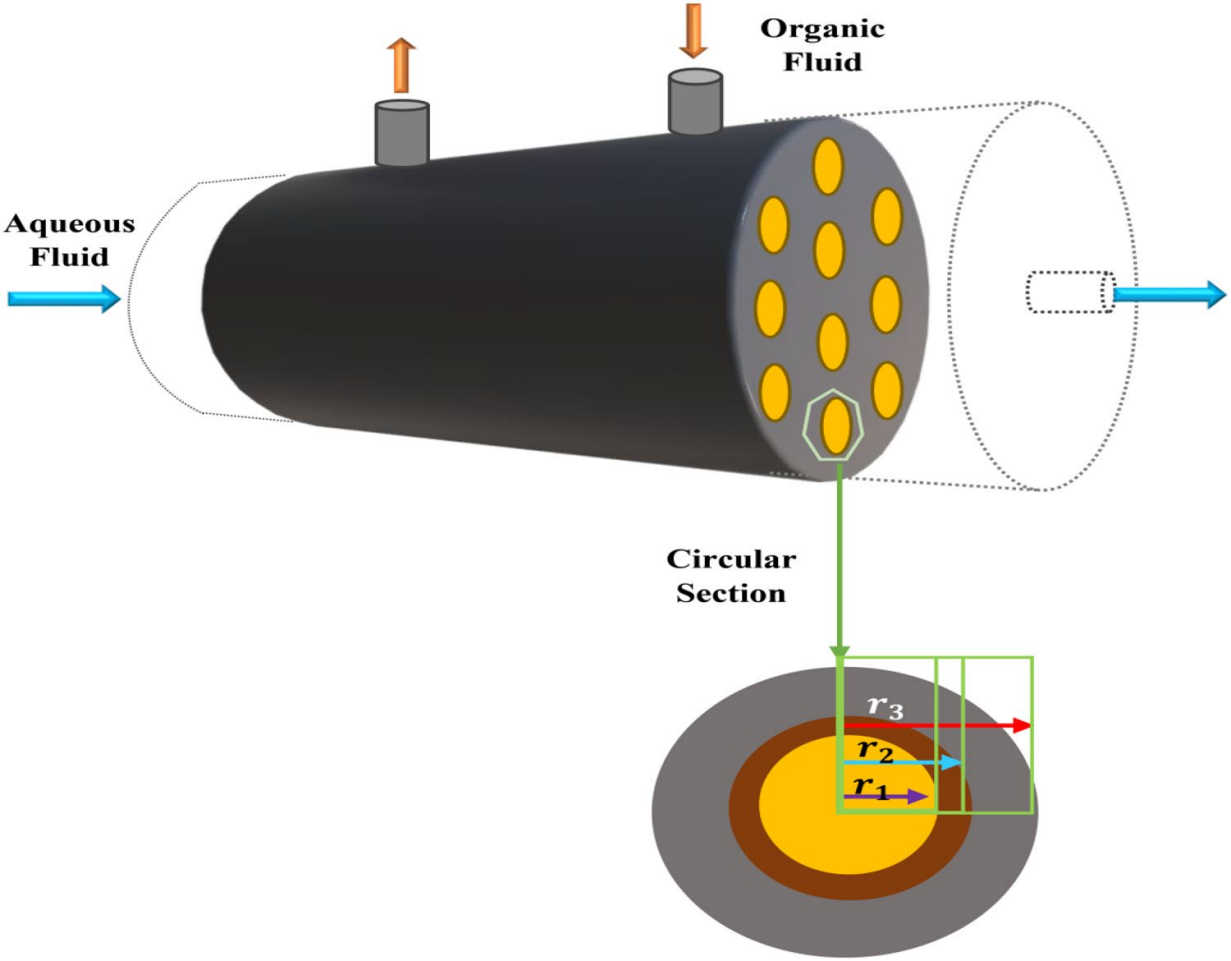
Schematic representation of membrane separation process technology studied in this work.

**Figure 2:**
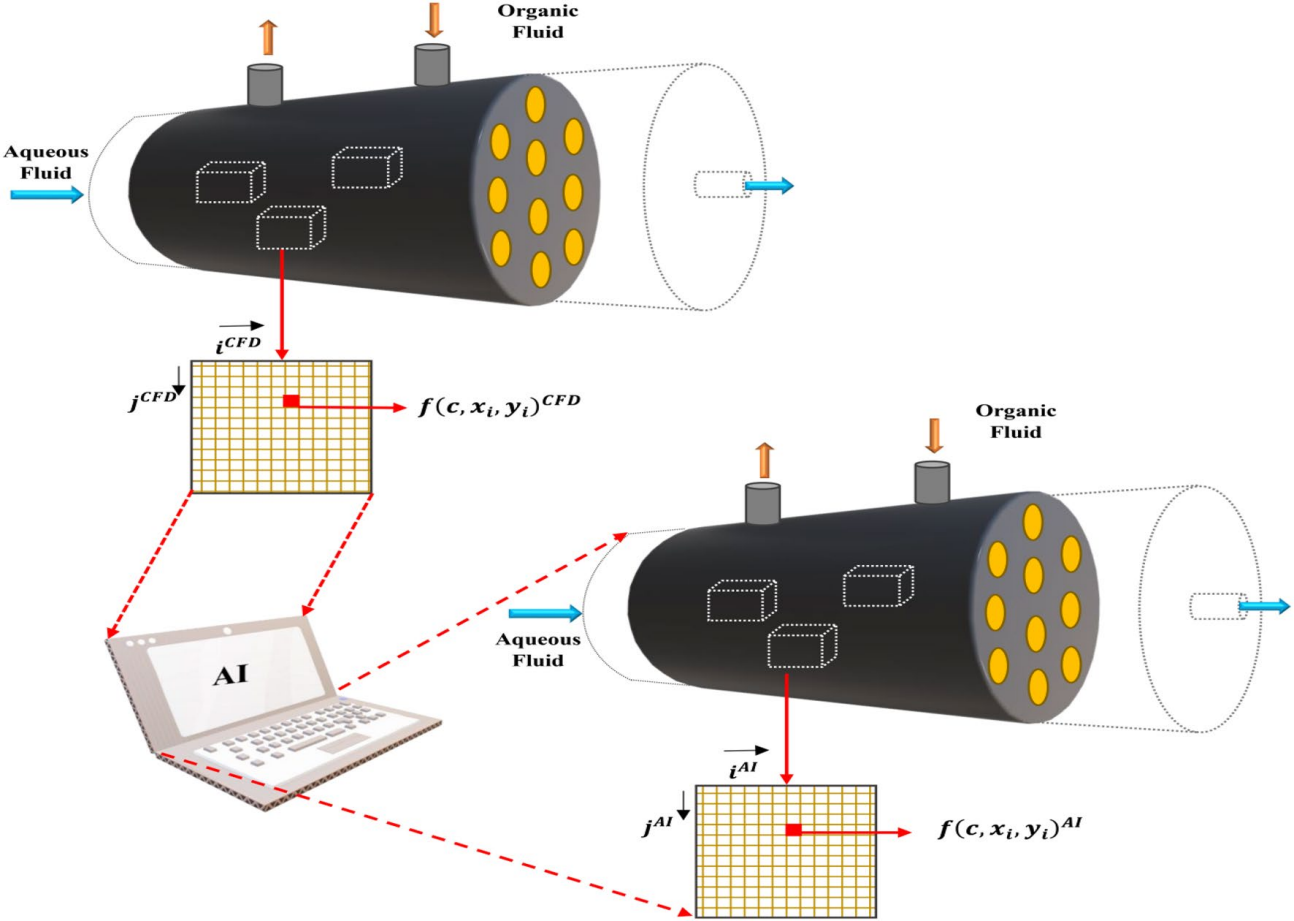
Schematics of CFD node connection with artificial intelligence node and prediction framework for membrane separation process technology.

Figure 1 shows a membrane contactor module, and from one side, the aqueous phase (feed) enters the module, and the organic fluid flows in the other side (shell side). Both phases are brought into contact using the membrane. As seen in the figure, the aqueous phase entered from different chambers to the membrane technology, and by the membrane technology, the chemical components can be separated from each other. For the separation solving process, the CFD was used; furthermore, the finite element method was utilized for the discretization of the complex Navier–Stokes equation formulas. By solving the Navier–Stokes equations, the approximate method of solving for the fluid concentration, the fluid velocity, and the fluid temperature could be accessed and calculated. Mass and momentum equations are computed in CFD simulation to represent component separation in the membrane structure. Equations to solve in the chamber are written, such as^20–22^:

1 $$D\left[ {\frac{{\partial^{2} C}}{{\partial r^{2} }} + \frac{1}{r}\frac{\partial C}{{\partial r}} + \frac{{\partial^{2} C}}{{\partial z^{2} }}} \right] = U\frac{\partial C}{{\partial z}}$$

2 $$\begin{aligned} & \rho \frac{\partial U}{{\partial t}} - \nabla \cdot \eta \left( {\nabla U + \left( {\nabla U} \right)^{T} } \right) + \rho \left( {U \cdot \nabla } \right)U + \nabla p = F \\ & \nabla \cdot U = 0 \\ \end{aligned}$$

where, *C* represents the concentration of species (mol/m^3^), and *U* shows the velocity distribution (m/s). $r$ and $z$ are geometry characteristics (m), *p* is pressure (Pa), and *F* is force (N). After the numerical method for solving the membrane technology, the CFD results were used in the training process of AI, and after that, the data were inserted in the fuzzy structure for the prediction process. As seen in Fig. 2, the CFD elements were added in the AI one by one. Each of the CFD elements includes the information from fluid concentration, and point by point position of the fluid. The data were inserted in AI and after the exact training of the data, a new domain was created that relates to the nodes of AI, and nodes of neural network. As seen in the figure, the nodes are very similar to CFD and again we have the separation in membrane technology when the aqueous fluid is inserted in the chambers, and from the other side, the organic fluid is inserted from one side and getting out of the system from another side.

## ANFIS method

**Figure 3:**
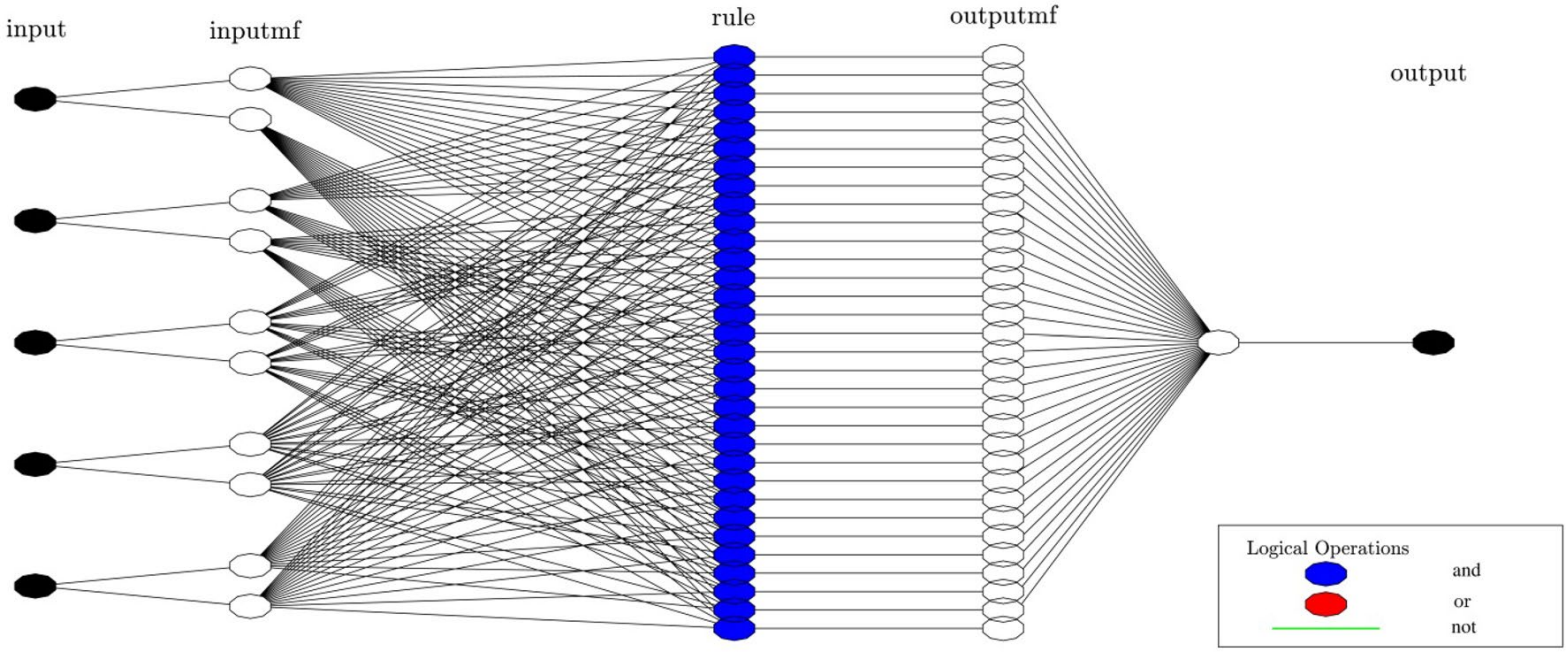
ANFIS structure, five inputs, number of MFs = 2.

ANFIS is a fuzzy inference system, precisely predicts the performance of nonlinear and complex systems^23–25^. Three different kinds of fuzzy reasoning exist where if–then rules proposed by Sugeno and Takagi are extensively run in ANFIS structure^26^. Figure 3 represents the structure of the ANFIS method for the estimation of the hydrodynamic features within the domain. The function of the ith rule is:

3 $${w}_{i}={\mu }_{Ai}\left(input1\right) {\mu }_{Bi}\left(input1\right){\mu }_{ci}(input1) {\mu }_{Di}(input1) {\mu }_{Ei}(input1)$$

in which *w*_*i*_ represents the signal out-coming from the node of the second layer. Moreover, *μ*_*Ai*_, *μ*_*Bi,*_* μ*_*Ci*_, *μ*_*Di*_ and *μ*_*Ei*_ indicate the signals incoming from MFs run on inputs, to the node of the 2nd layer. Within the 3rd layer, the relative value of each rule relating to the firing strength is determined, which is equivalent to the weight of each layer over the whole quantity of firing strengths of all rules:

4 $$\stackrel{-}{{w}_{i}}=\frac{{w}_{i}}{\sum \left({w}_{i}\right)}$$

where $\stackrel{-}{{w}_{i}}$ denotes the normalized firing strengths. Layer 4 employed the function of a consequence if–then rule suggested by Sugeno and Takagi^26^.

Therefore, the node function is:

5 $$\stackrel{-}{{w}_{i}}{f}_{i}=\stackrel{-}{{w}_{i}}({p}_{i}(input1)+{q}_{i}(input2)+{r}_{i}(input3)+{s}_{i}(input4)+{t}_{i}(input5)+{u}_{i})$$

in which *p*_*i*_, *q*_*i*_, *r*_*i,*_ , *s*_*i,*_ , *t*_*i,*_ and *u*_*i*_ represent the if–then parameter rules, which are called consequent parameters. ${f}_{i}$ can be also considered as output results and function of model. The signals incoming from the forth layer are combined to attain the model output representing the estimation outcome. Detailed description of ANFIS model has been reported elsewhere^27^.

## Results and discussion

**Figure 4:**
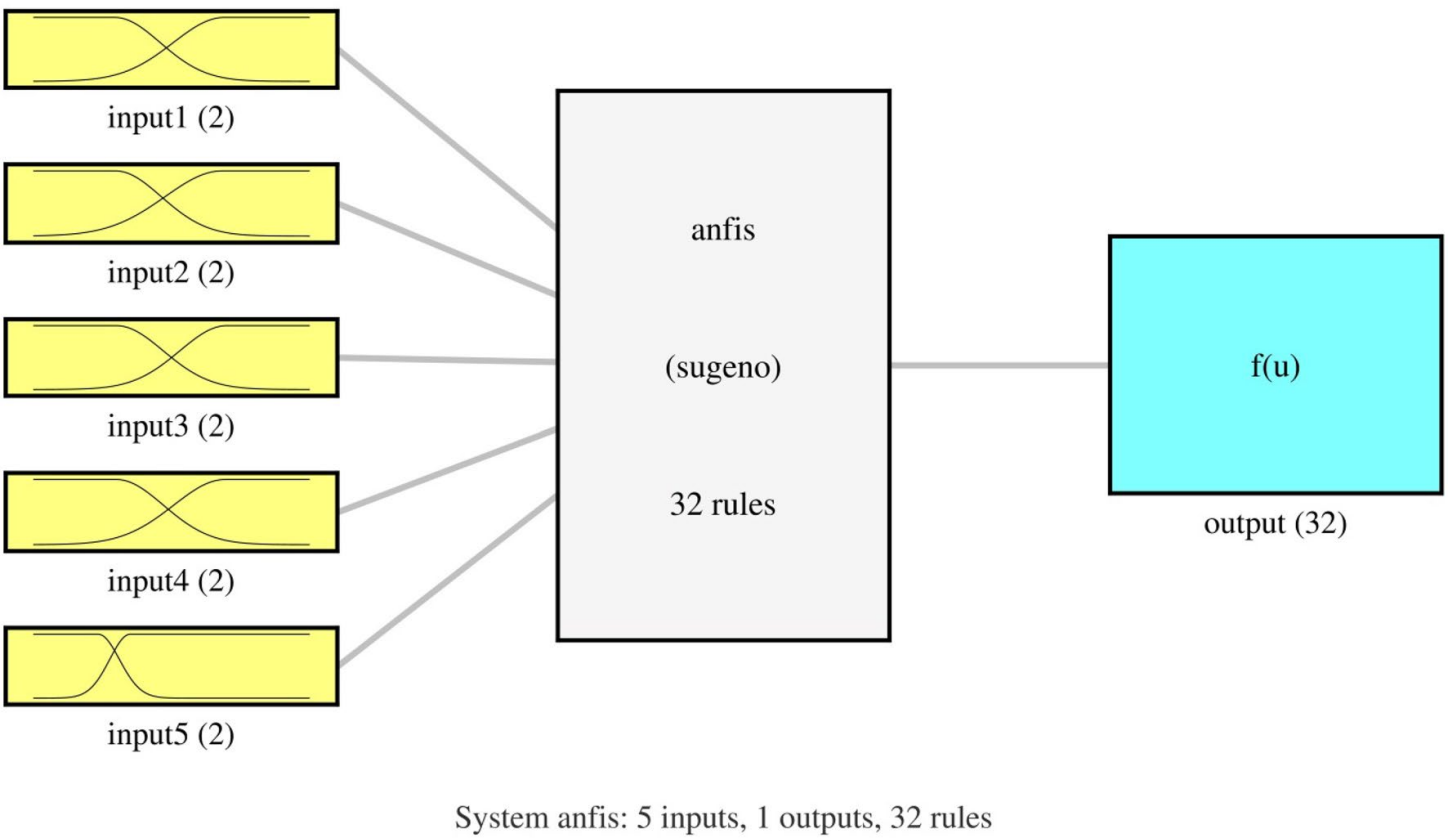
System ANFIS, five inputs, one output, 32 rules.

In this study, five parameters as input and one parameter as output have been considered for the ANFIS method. Parameter T (K), weight, p_co_ (kpa), AARD, and alfa represent the 1st–5th input of the ANFIS method, respectively, with the prediction of species concentration representing as the ANFIS method output (see Fig. 4).

**Figure 5:**
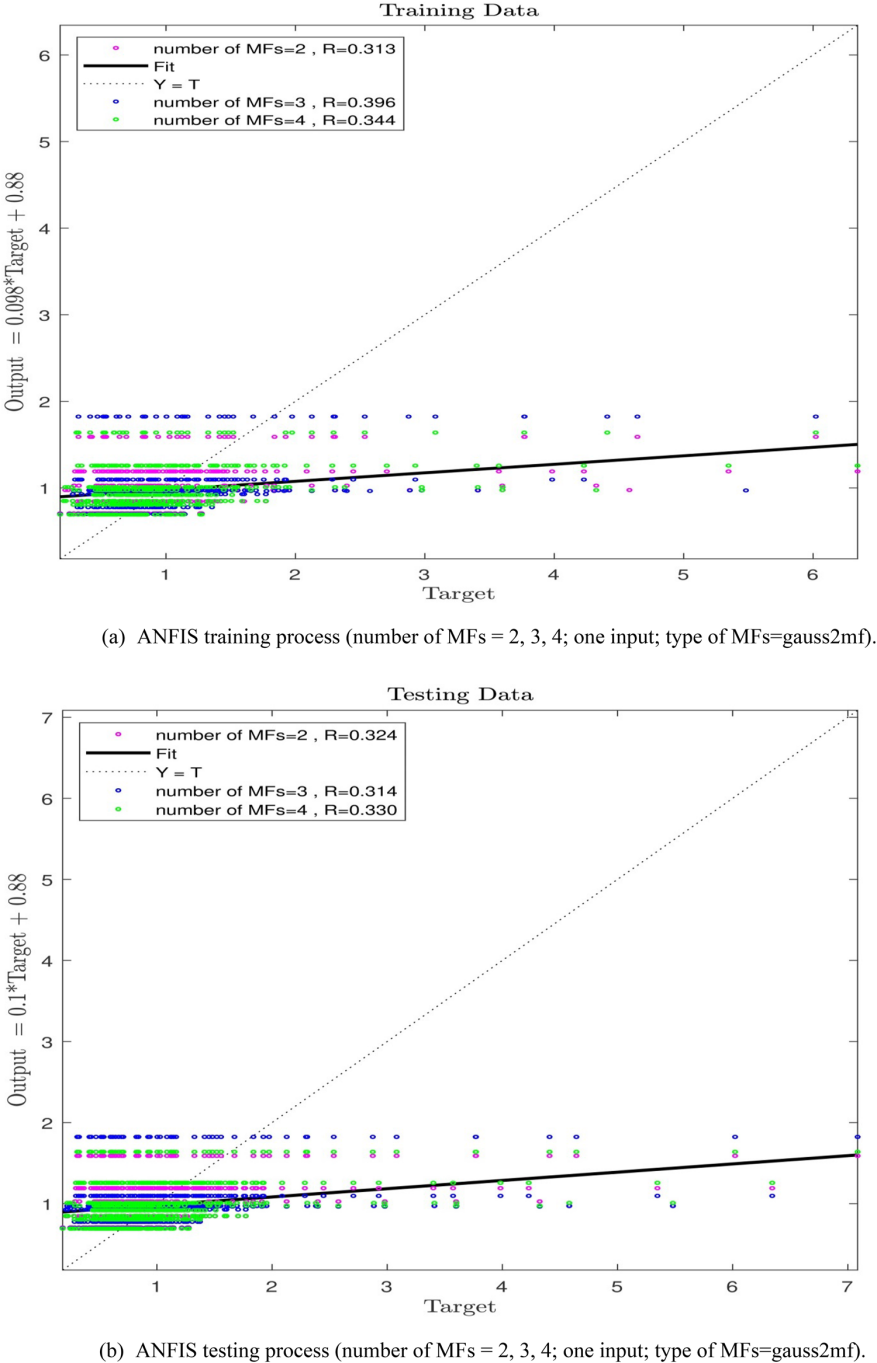
(**a**) ANFIS training process (number of MFs = 2, 3, 4; one input; type of MFs = gauss2mf). (**b**) ANFIS testing process (number of MFs = 2, 3, 4; one input; type of MFs = gauss2mf).

In this study, a number of maximum 500 iterations are assumed, and the P which is indicative of the number of data participating in training process shall be as equal to 60%; the membership functions (MFs) in the present study is considered to be gauss2mf (Gaussian combination membership function). The learning process was evaluated, considering one input to examine the procedure of ANFIS capability. When the number of MFs is equal to 2, then regression(R) shall be equal to 0.31, indicating 31% intelligence achievement by the ANFIS method. In order to increase the ANFIS intelligence level, the increase in the number of MFs was assessed and in accordance with Fig. 5a,b, the R-value for the MFs = 3 shall be equal to 0.39 and if MFs = 4, then the R-value will reduce to 0.34, showing that a higher number of MFs will produce the minute effect on the increase in the ANFIS intelligence level. Accordingly, the increase in the number of inputs was evaluated. Given the number of MFs = 2, the increase in R-value to 0.69, demonstrates an increase of 38% in ANFIS intelligence compared to the conditions where the input value was equal to 1.

When the number of inputs was low in the model, the regression was low and lower than 0.32, but by increasing the number of inputs, the AI algorithm led to an increase in the prediction capability of the model. Therefore, the model could complete the prediction with better accuracy. When the number of inputs increased to 4, the R-value reached 0.99, showing that the high accuracy of the model as well as its high capability in prediction.

**Figure 6:**
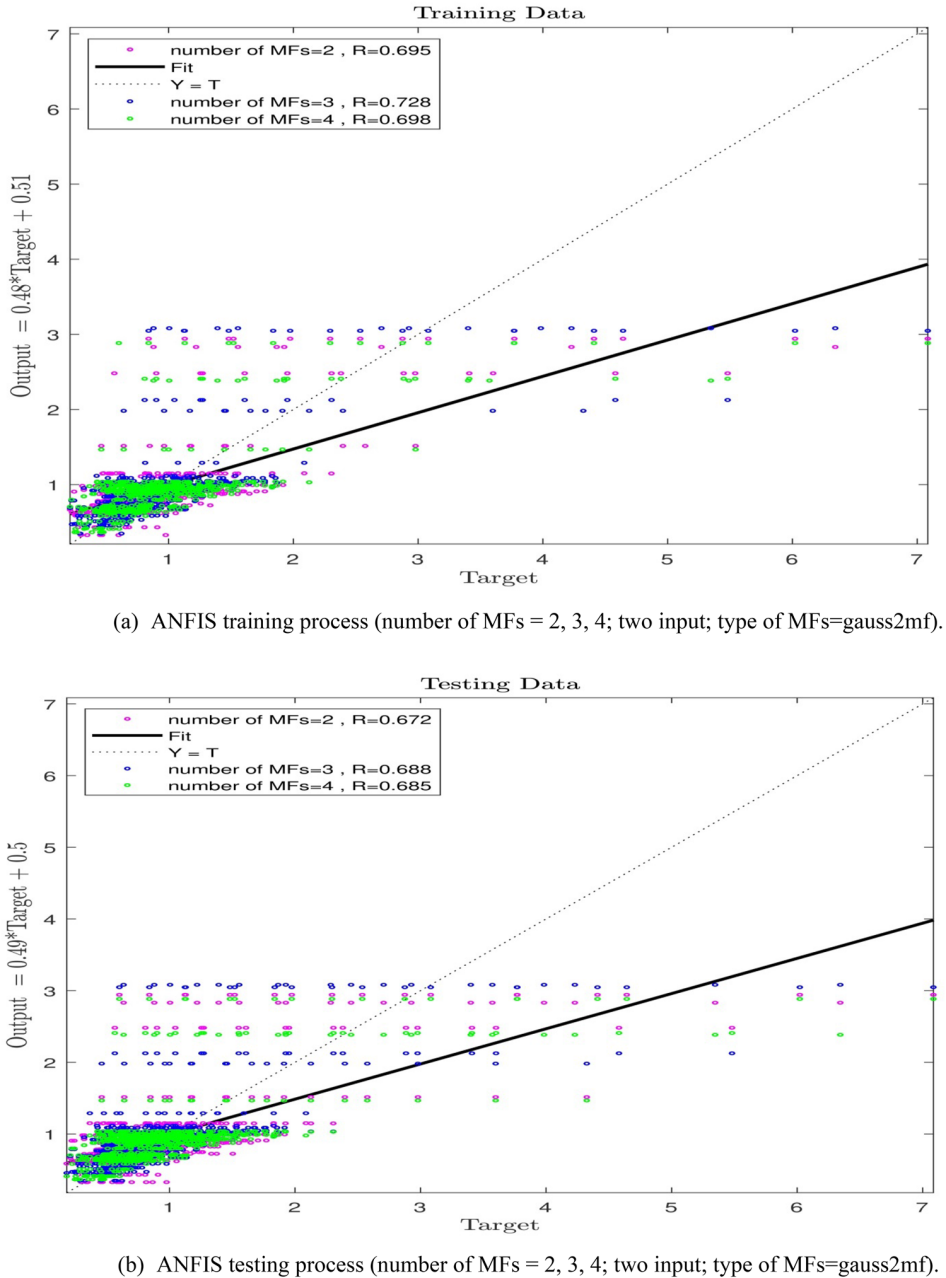
(**a**) ANFIS training process (number of MFs = 2, 3, 4; two input; type of MFs = gauss2mf). (**b**) ANFIS testing process (number of MFs = 2, 3, 4; two input; type of MFs = gauss2mf).

This increase in the ANFIS is not sufficient to reach a complete intelligence, and to fulfill this goal, further increase in the number of MFs to 3 and 4 was again investigated. As per the Fig. 6a,b, the R-value for MF = 3 shall be equal to 0.72, and for the number of MF = 4, and the R-value is 0.69 (R = 0.69), which is the indicative of the fact that given the study obtained data, the increase in the number of MFs is not effective in increasing the ANFIS prediction capability.

**Figure 7:**
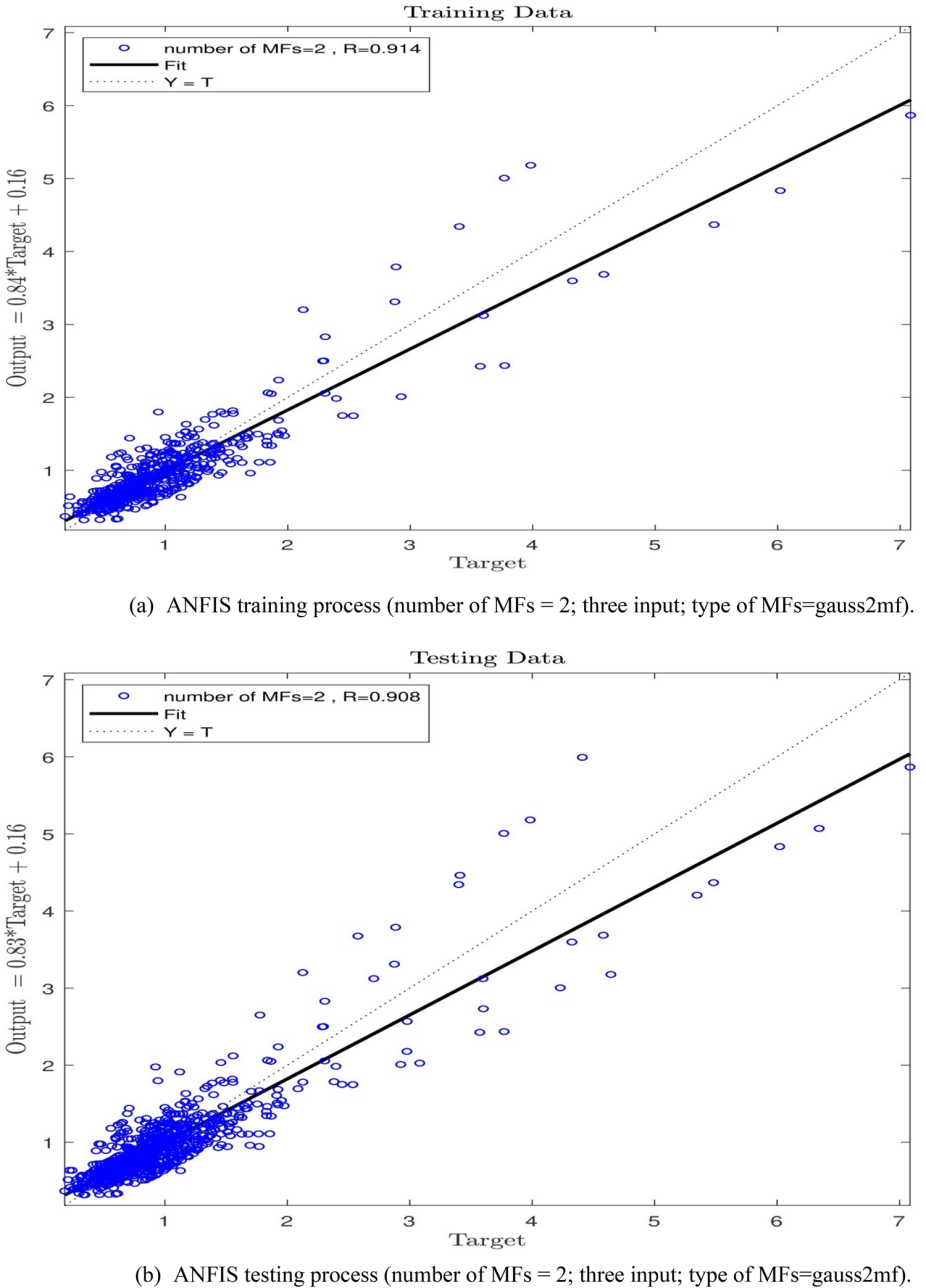
(**a**) ANFIS training process (number of MFs = 2; three input; type of MFs = gauss2mf). (**b**) ANFIS testing process (number of MFs = 2; three input; type of MFs = gauss2mf).

**Figure 8:**
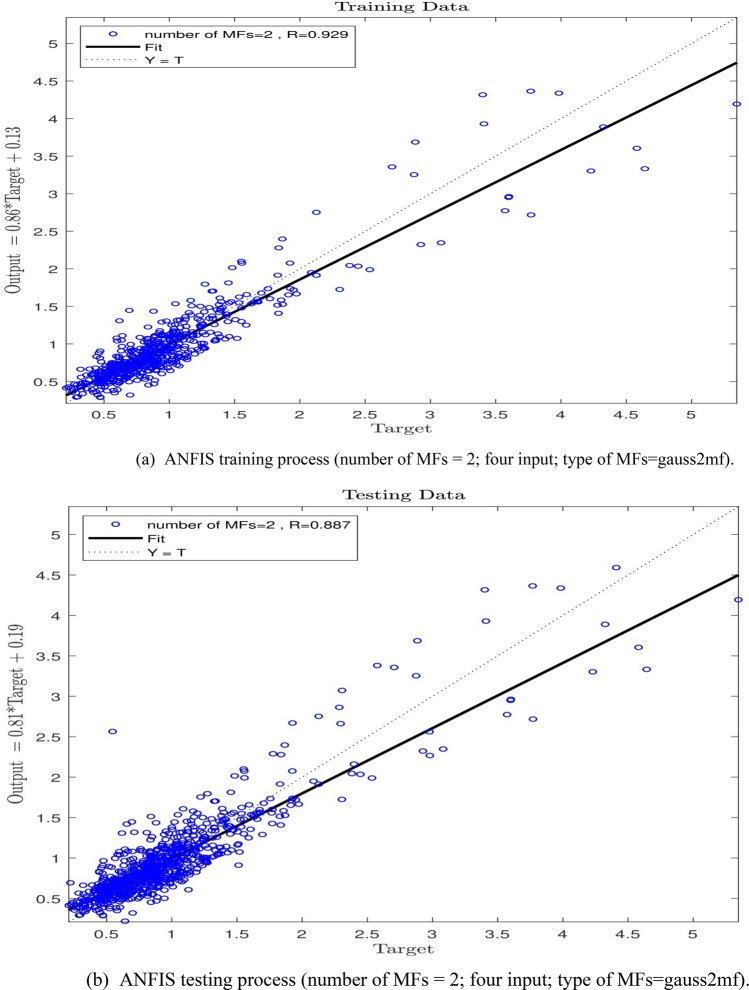
(**a**) ANFIS training process (number of MFs = 2; four input; type of MFs = gauss2mf). (**b**) ANFIS testing process (number of MFs = 2; four input; type of MFs = gauss2mf).

**Figure 9:**
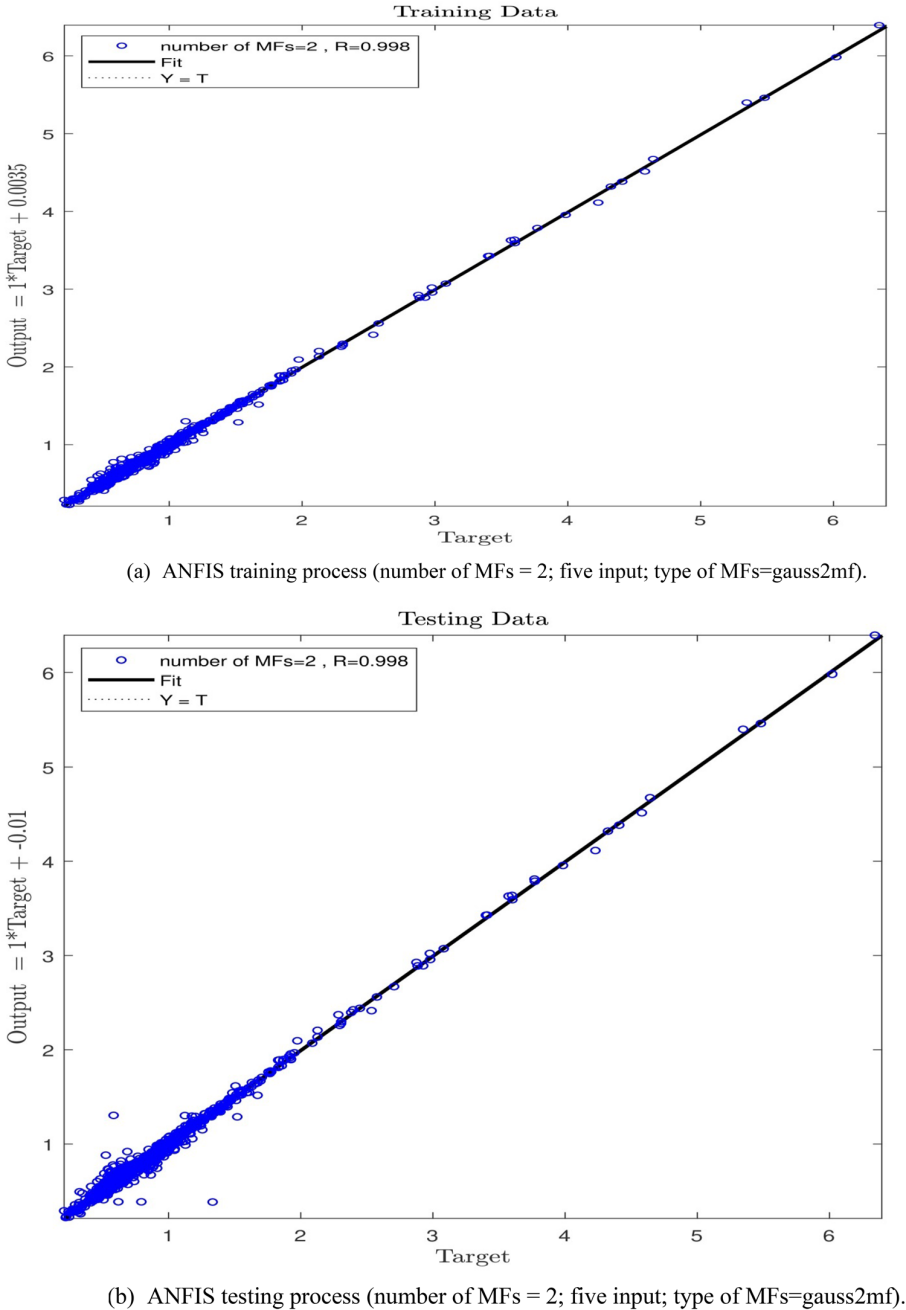
(**a**) ANFIS training process (number of MFs = 2; five input; type of MFs = gauss2mf). (**b**) ANFIS testing process (number of MFs = 2; five input; type of MFs = gauss2mf).

In the next stage of the study, an increase from 2 to 3 in the number of inputs with a MFs value of 2 was observed. As Fig. 7a,b show, the system has witnessed an appropriate value of the intelligence in training and testing processes, and the R-value for the testing process has been equal to 0.90, which means an ANFIS intelligence value of 90%. Since an increase in the number of MFs was not extensively effective in increasing the ANFIS prediction capability, the number of inputs was increased to 4, and the learning processes were performed for the number of MFs = 2. The findings, according to Fig. 8a,b are not indicative of any increase in the system intelligence compared with the conditions where the number of inputs is equal to 3. Hence, the number of inputs was increased to 5, and the training–testing processes were conducted with the number of MFs = 2. According to Fig. 9a,b a wonderful increase in the amount of ANFIS intelligence can be observed so that an R-value of 0.99 and 0.94 for training and testing respectively shows a sharp increase in the amount of ANFIS method intelligence to 94% level.

**Figure 10:**
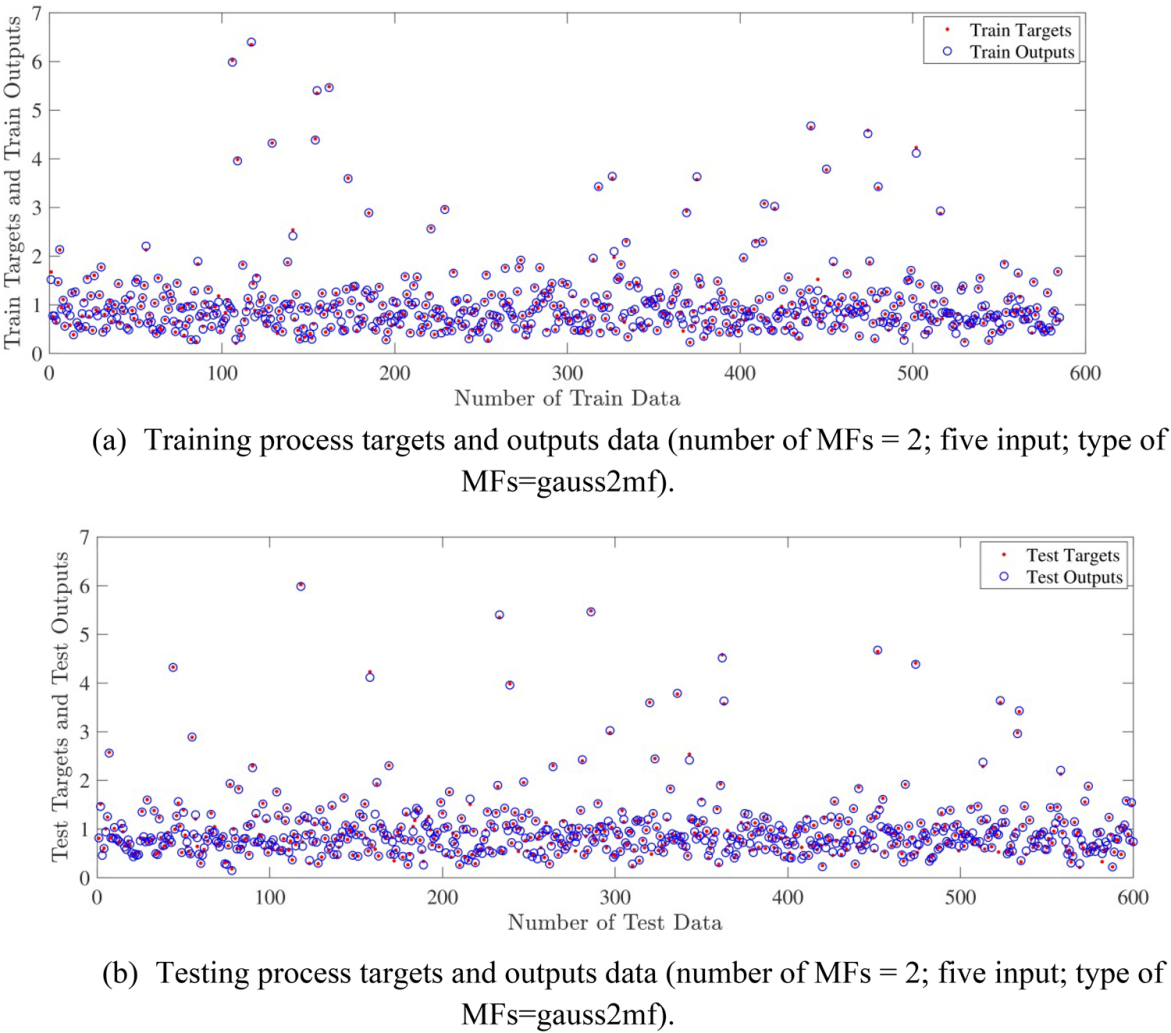
(**a**) Training process targets and outputs data (number of MFs = 2; five input; type of MFs = gauss2mf). (**b**) Testing process targets and outputs data (number of MFs = 2; five input; type of MFs = gauss2mf).

**Figure 11:**
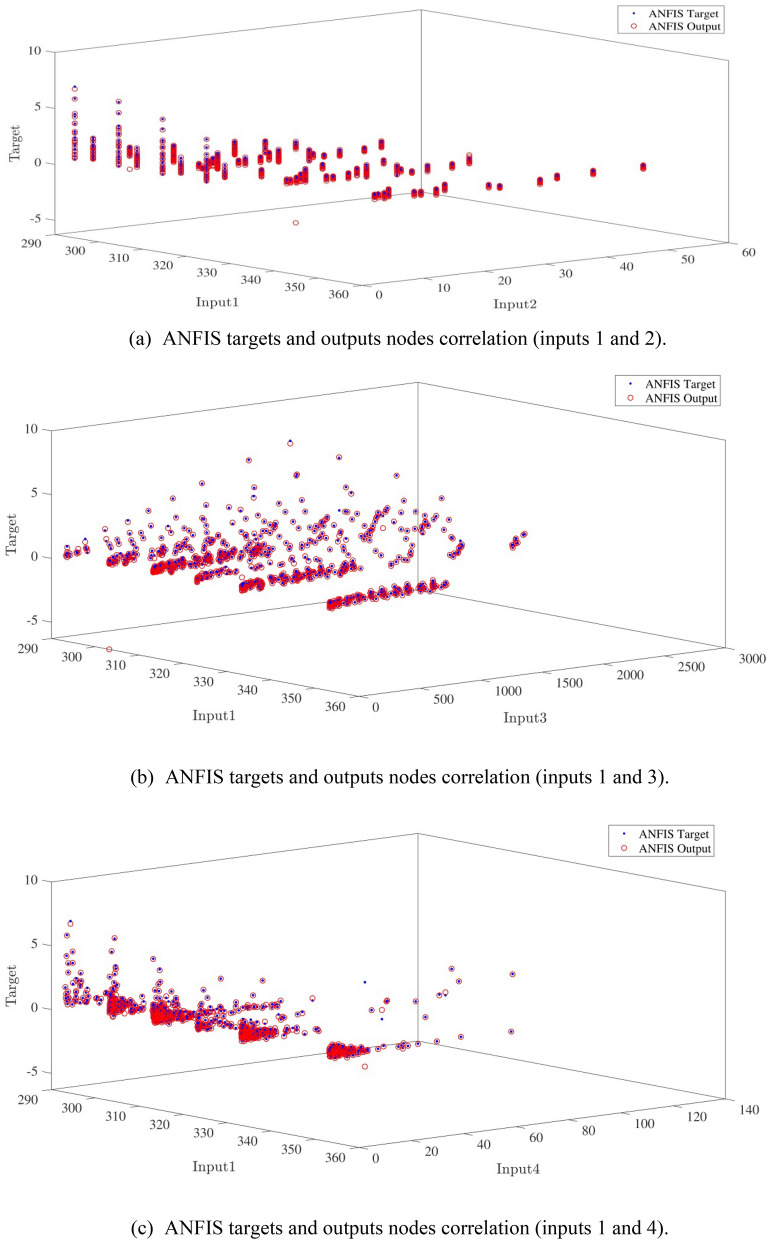

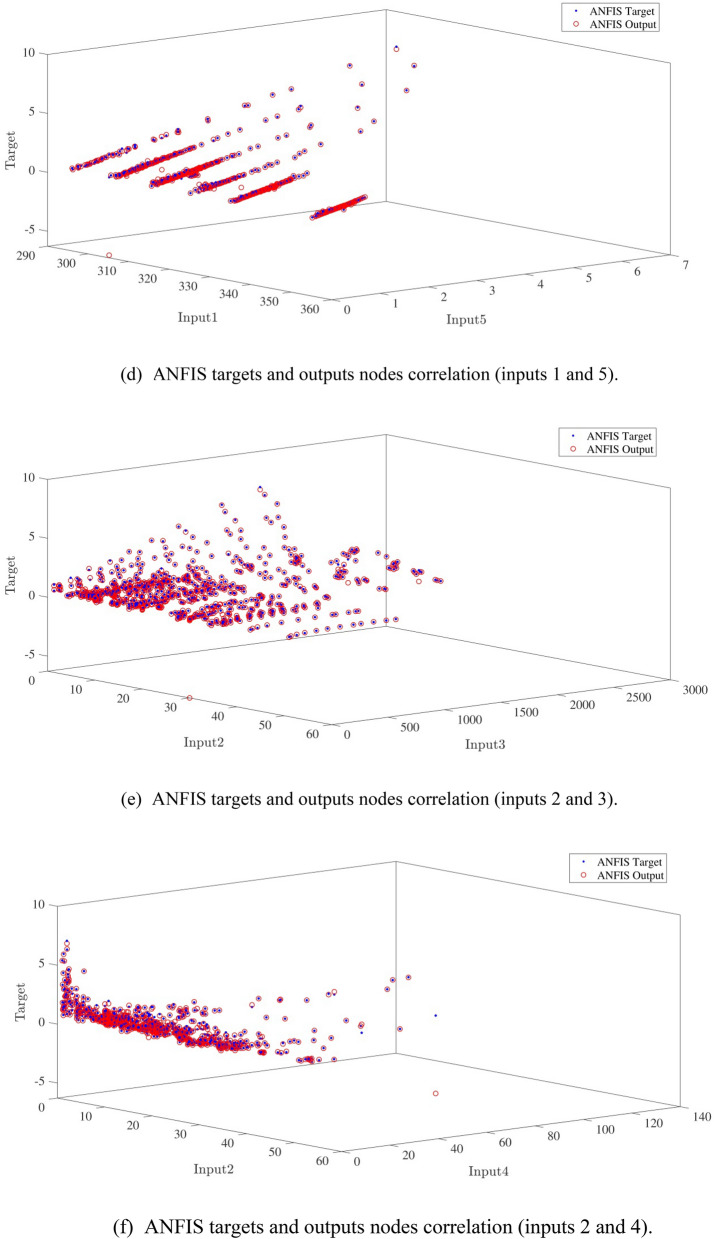

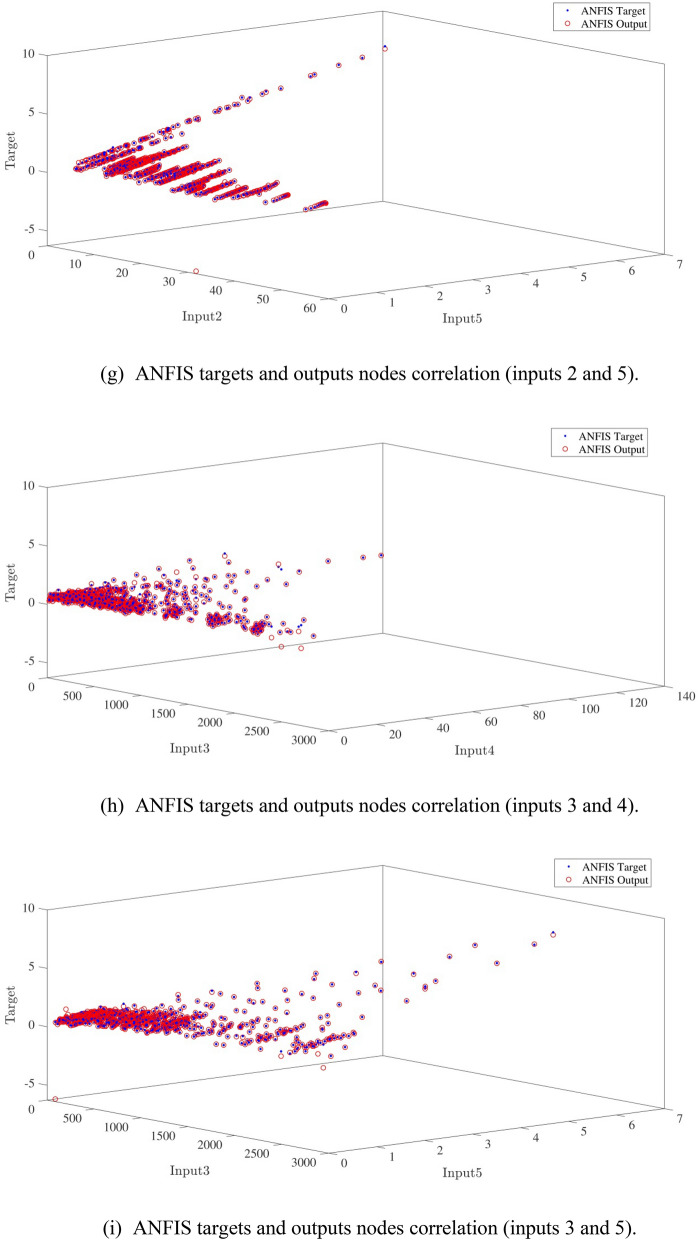

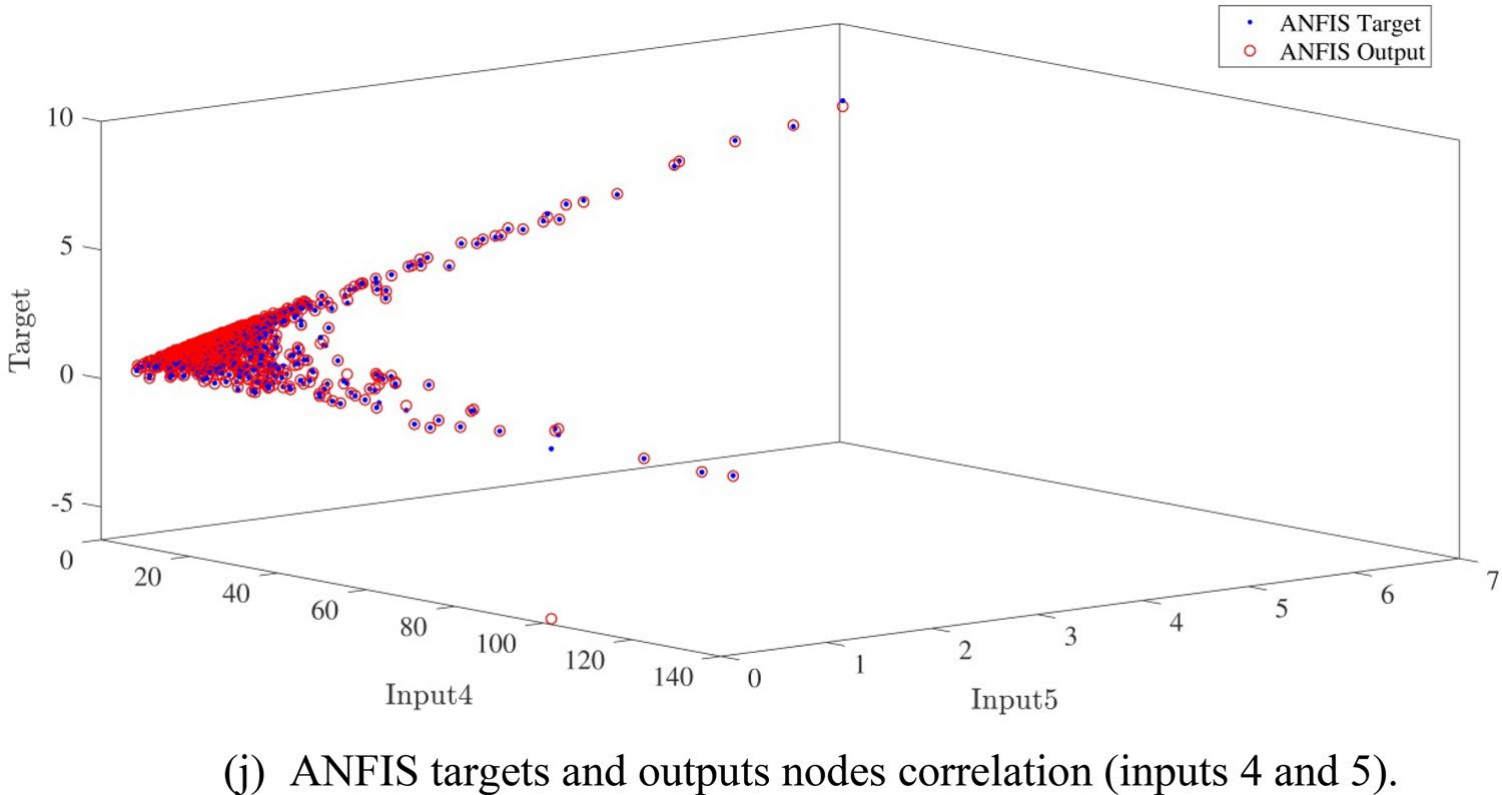
(**a**) ANFIS targets and outputs nodes correlation (inputs 1 and 2). (**b**) ANFIS targets and outputs nodes correlation (inputs 1 and 3). (**c**) ANFIS targets and outputs nodes correlation (inputs 1 and 4). (**d**) ANFIS targets and outputs nodes correlation (inputs 1 and 5). (**e**) ANFIS targets and outputs nodes correlation (inputs 2 and 3). (**f**) ANFIS targets and outputs nodes correlation (inputs 2 and 4). (**g**) ANFIS targets and outputs nodes correlation (inputs 2 and 5). (**h**) ANFIS targets and outputs nodes correlation (inputs 3 and 4). (**i**) ANFIS targets and outputs nodes correlation (inputs 3 and 5). (**j**) ANFIS targets and outputs nodes correlation (inputs 4 and 5).

**Figure 12:**
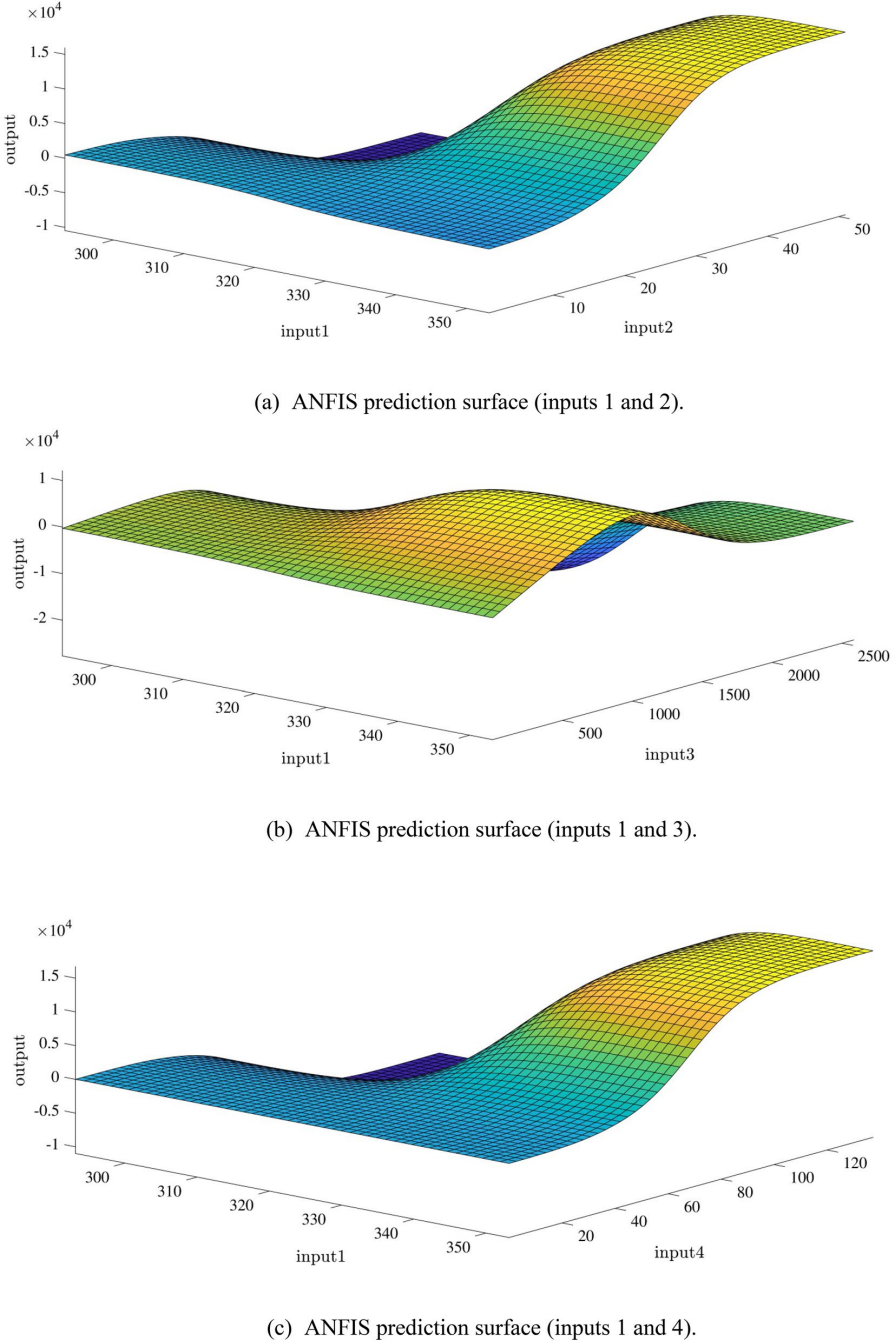

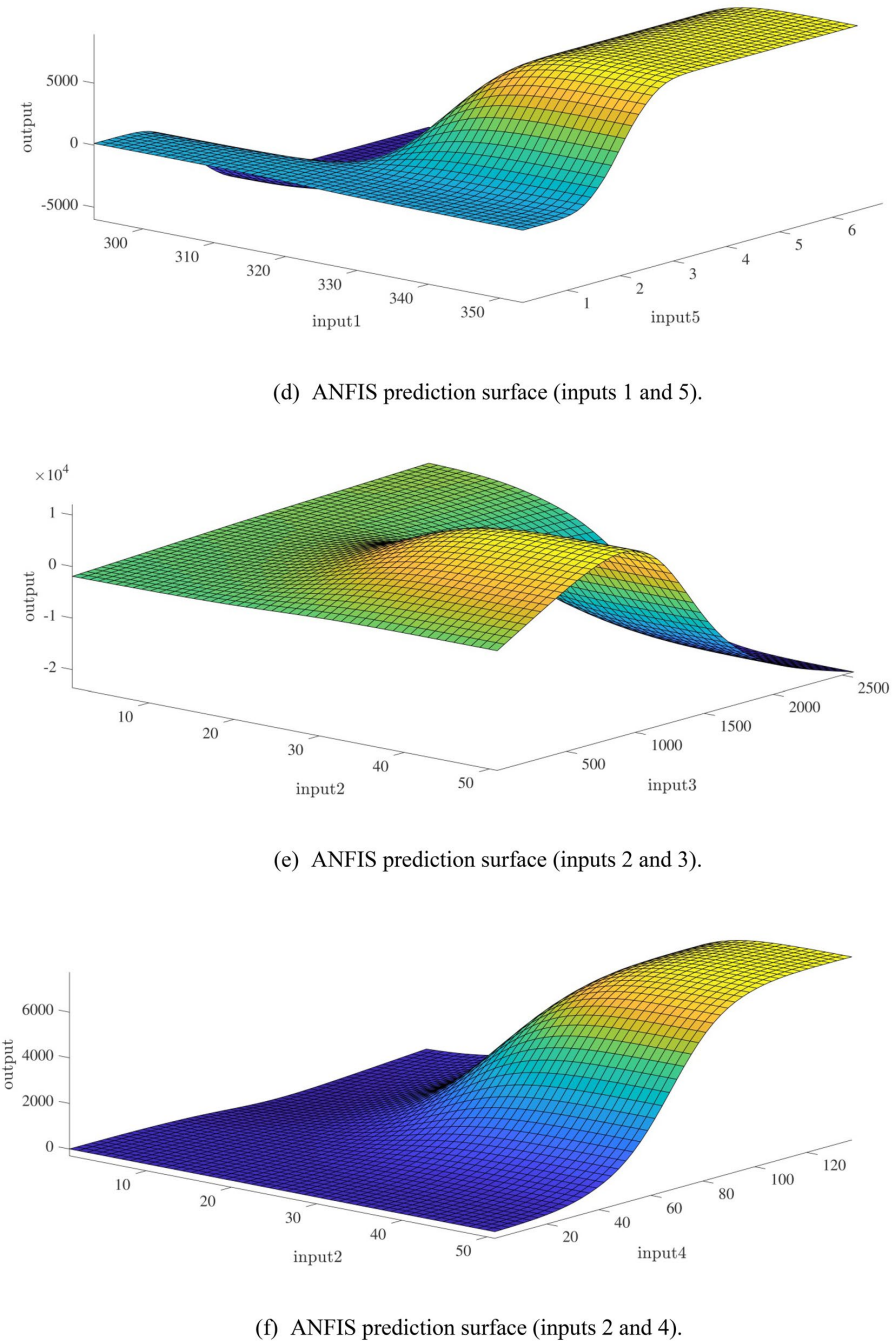

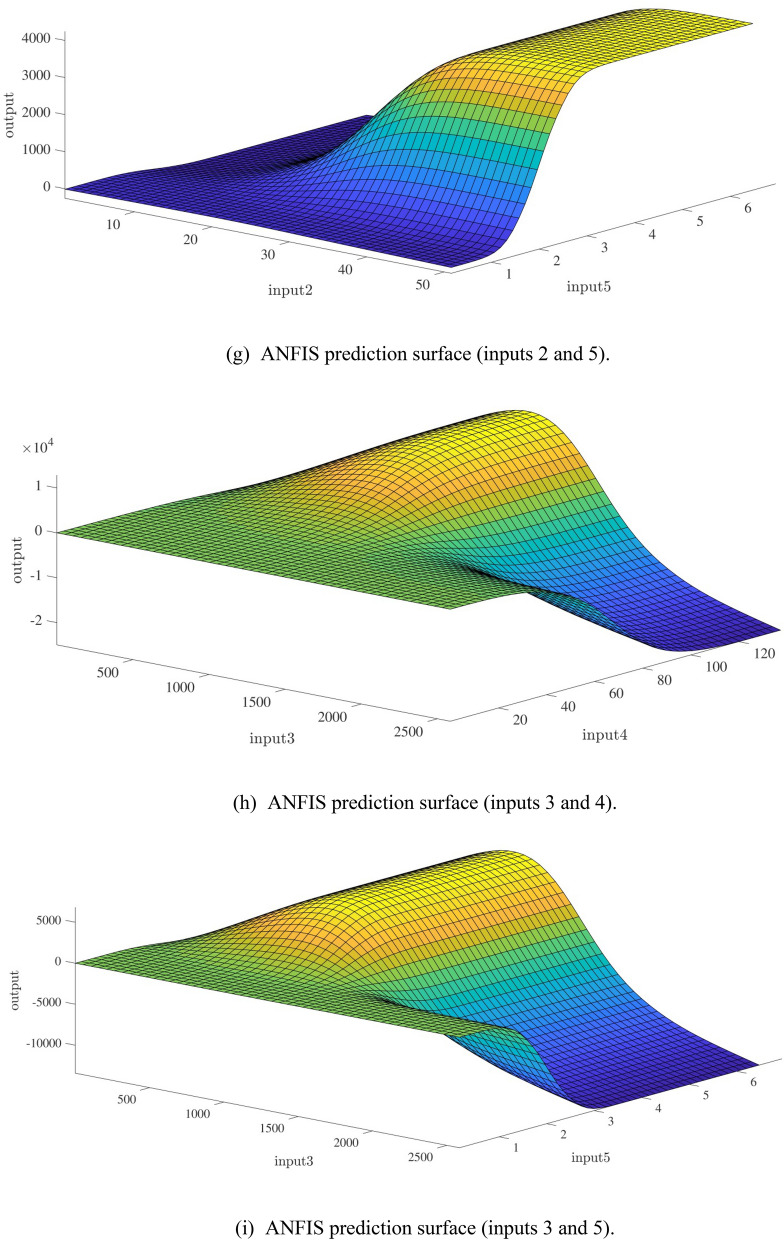

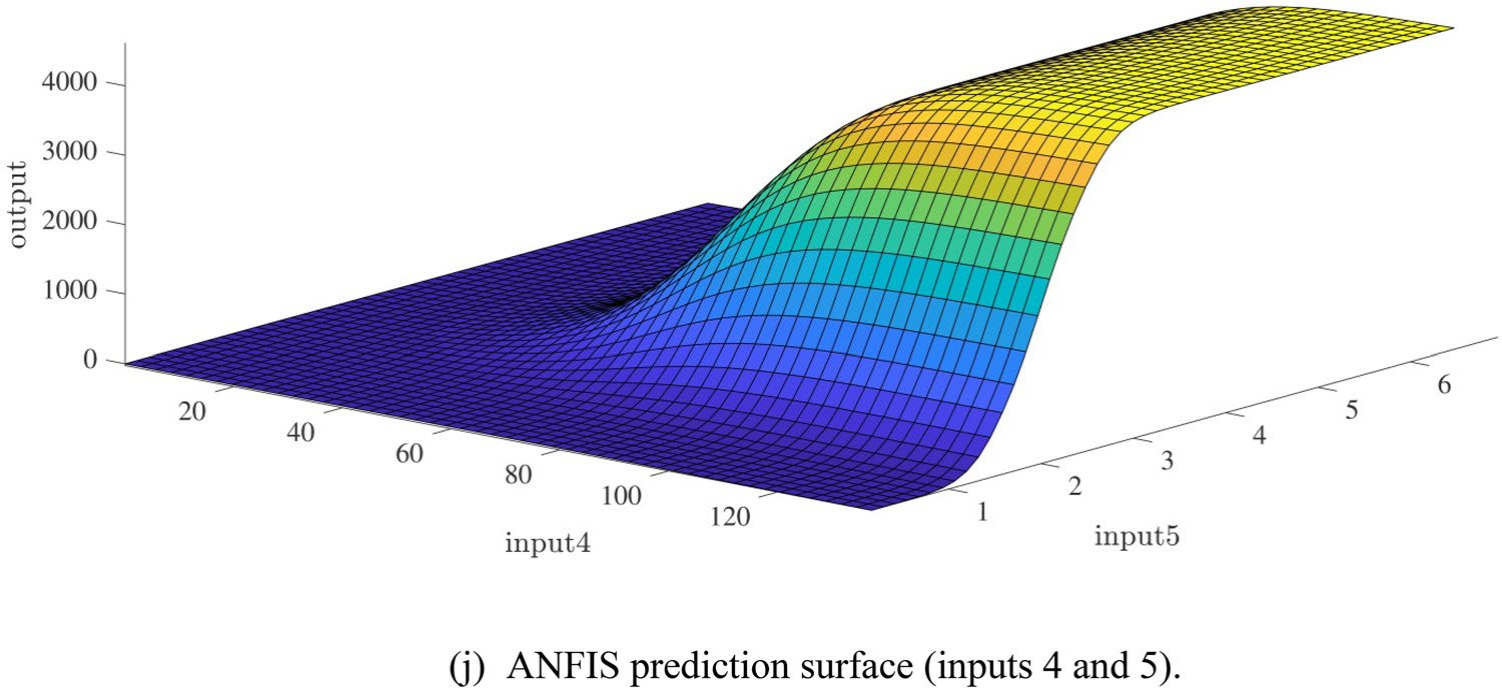
(**a**) ANFIS prediction surface (inputs 1 and 2). (**b**) ANFIS prediction surface (inputs 1 and 3). (**c**) ANFIS prediction surface (inputs 1 and 4). (**d**) ANFIS prediction surface (inputs 1 and 5). (**e**) ANFIS prediction surface (inputs 2 and 3). (**f**) ANFIS prediction surface (inputs 2 and 4). (**g**) ANFIS prediction surface (inputs 2 and 5). (**h**) ANFIS prediction surface (inputs 3 and 4). (**i**) ANFIS prediction surface (inputs 3 and 5). (**j**) ANFIS prediction surface (inputs 4 and 5).

Figure 10a,b represent a comparison between the output and target data pertaining to the ANFIS method for training and testing processes. Also, Fig. 11a–j illustrate a good consistency between the ANFIS target and ANFIS output considering various input values. This constitutes a significant qualification of the artificial intelligence thanks to which one can predict the points which were absent in the learning process(see Fig. 12a–j).

**Figure 13:**
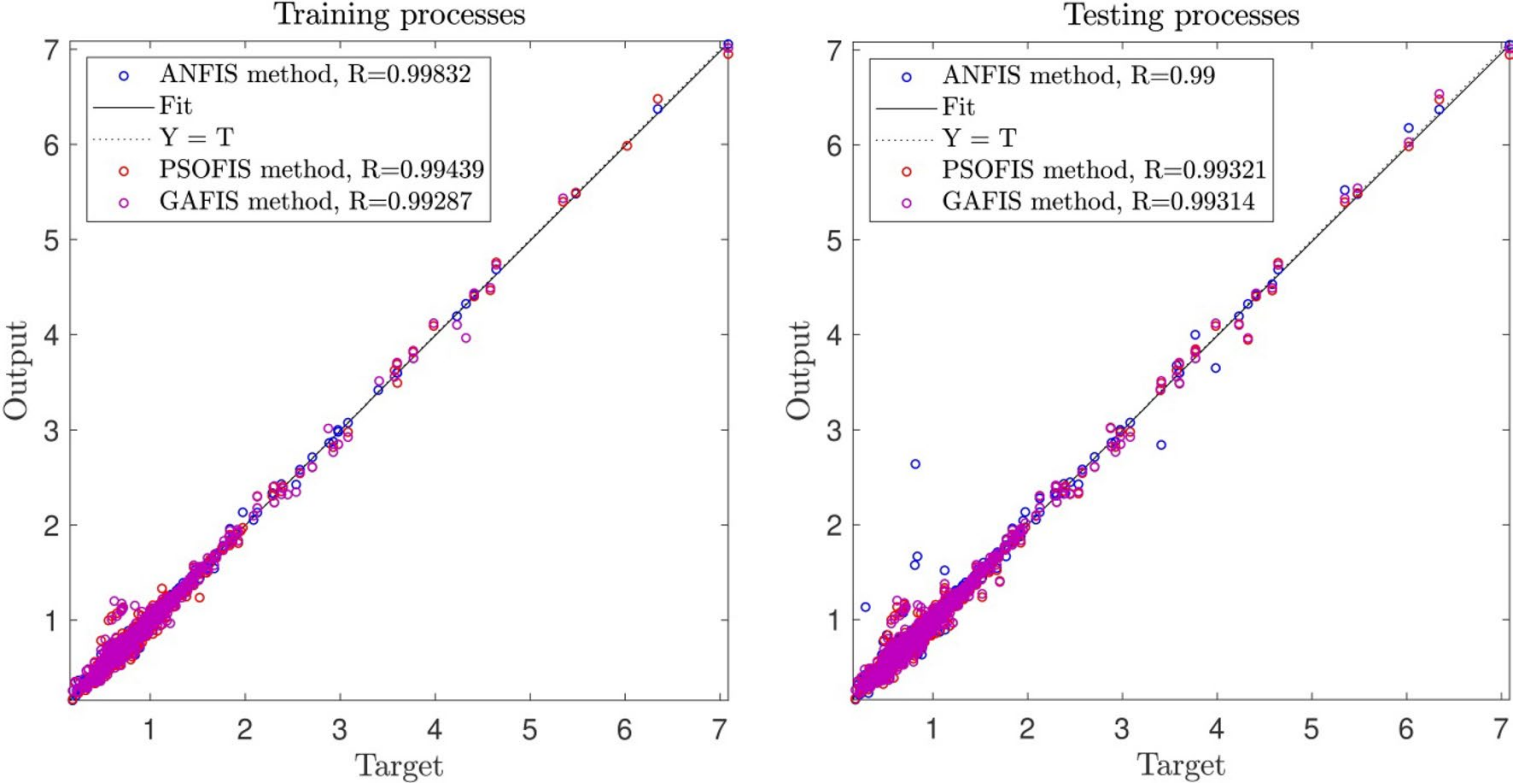
Learning processes of the highest correlation coefficient results using ANFIS and particle swarm optimization (PSO) algorithm and genetic algorithm (GA) which used as trainer in fuzzy inference system that called PSOFIS and GAFIS respectively.

**Figure 14:**
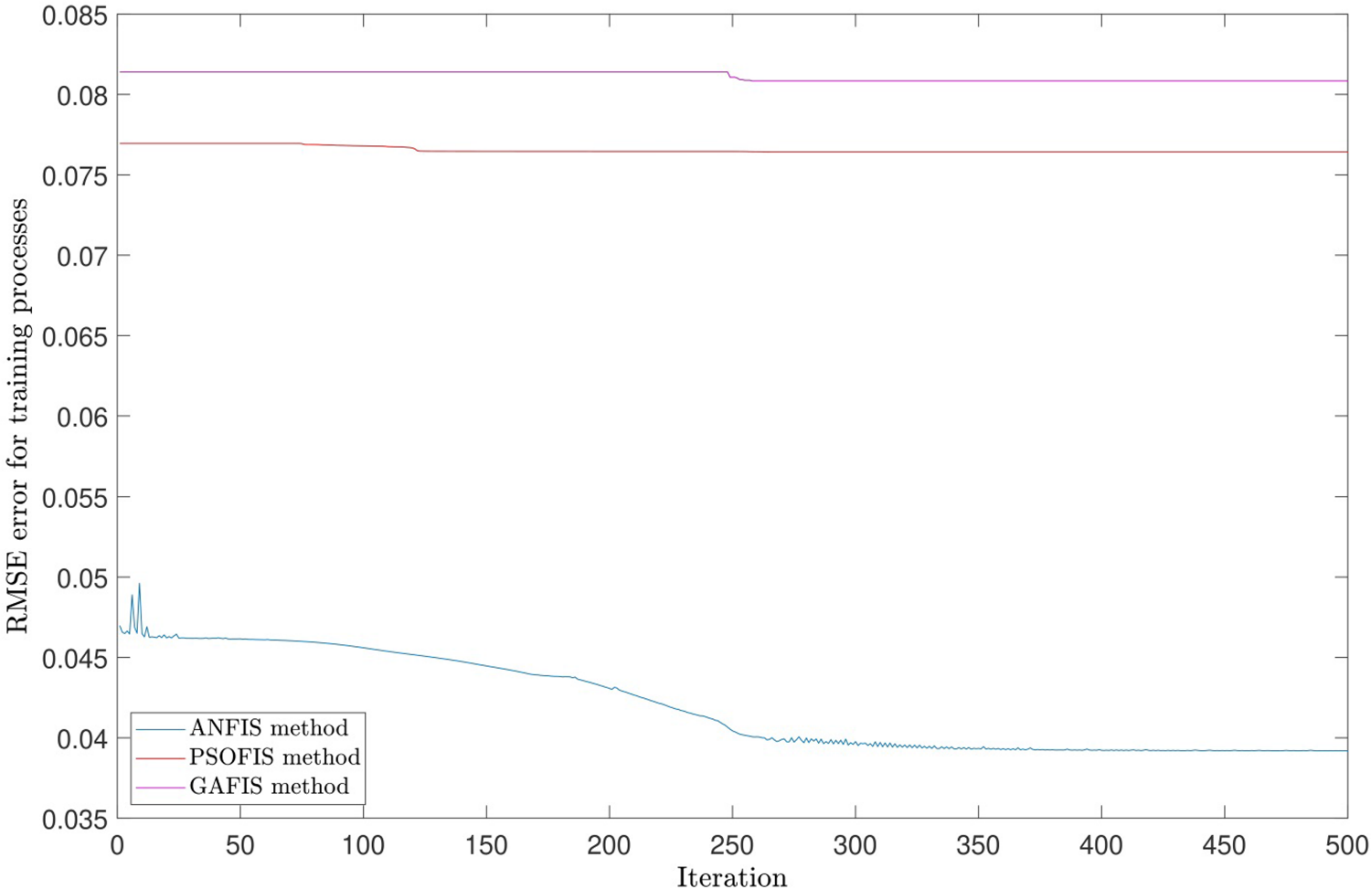
Comparison of RMSE error patterns for ANFIS, PSOFIS, GAFIS methods based on iteration.

The obtained results were studied with different methods to investigate the capacity of the model for the prediction process. The ANFIS method was compared to Particle Swarm Optimization (PSOFIS) and Genetic Algorithm (GAFIS), and as shown in Fig. 13, the results in testing and training were in agreement, and they were investigated regarding their accuracy. The figure showed that the results in both of the processes have a high accuracy showing the high capability of the model. The methods also were compared regarding their error, and as shown, ANFIS error, according to R-value, was the lowest among the two other methods, which were PSOFIS and GAFIS methods though they were in a suitable range of accuracy (see Fig. 14). The methods were studied regarding the error, time of prediction, and training. The results showed that the methods had a high capability in prediction, and they could predict a process very fast. All the data for the dataset could complete the training process in less than 103 s, and the prediction process could be completed in less than 2 s showing the high capability of the methods in the prediction process. Furthermore, as shown in Table 1, the error in testing and training processes were studied. The table also revealed that the three methods had high accuracy and capability in prediction.

**Table 1:** Training and testing errors, learning and prediction times for the best learned FIS of ANFIS, PSOFIS and GAFIS methods.

Methods	ANFIS	GAFIS	PSOFIS
Number of inputs	5	5	5
Maximum of iteration	500	500	500
Percentage of P	70	70	70
Clustering type	Grid partition	Subtractive clustering	Subtractive clustering
Training MSE error	0.001535665	0.006535045	0.005841158
Training RMSE error	0.039187567	0.080839622	0.076427468
Training mean error	1.86015E − 06	− 0.00145448	0.002179109
Training standard deviation (StD)	0.039221103	0.080895708	0.076461776
Training correlation coefficient (R) error	0.99832132	0.992869298	0.994394045
Training coefficient of determination (R^2^) error	0.996645458	0.985789443	0.988819516
Testing MSE error	0.009672048	0.00655753	0.006493703
Testing RMSE error	0.098346572	0.080978575	0.080583516
Testing mean error	− 0.007010998	0.000766209	0.002871209
Testing standard deviation (StD)	0.098155074	0.081023424	0.080580558
Testing correlation coefficient (R) error	0.989996681	0.993137387	0.99320752
Testing coefficient of determination (R^2^) error	0.980093427	0.98632187	0.986461178
Learning time (s)	102.6507035	85.5361454	57.1184928
Prediction time (s)	0.7148131	1.267197	0.1744804

As shown in the results, when one input was used, the error of the system and its accuracy were high, and the regression was about 0.32, which is not a suitable accuracy for an AI model. One of the parameters used in the study was the number of inputs, and by increasing it, the AI algorithm reached high capability for the prediction of the results. When the number of inputs increased to 5, the regression reached to 0.99. For studying the elements engaged in the prediction of a process in AI, the model needed to be trained with different parameters to see which input could be trained and achieved high capability in prediction. After that, the specific number of inputs could be used for the prediction. Sometimes, the models are complex, and a high number of inputs are needed to provide more and meaningful relationships between inputs and outputs. Therefore, the regression value and accuracy of the system increased, and a process could be predicted.

## Conclusion

In this study, the membrane technology was simulated via the finite element CFD method, and the data from CFD was studied in the AI algorithm. A new model was proposed by using AI that can model the separation process in the membrane system. This method could create a new domain of prediction in the AI framework by using the CFD data. The data from AI showed that AI reached its intelligence, and the system sent the intelligent signals when the maximum number of inputs was added to it. For example, when five inputs were added to the system, the system showed its best level of prediction, and it became fully predictive tools. When 1 or 2 inputs were used in the training process, the system did not have complete level of prediction, and they did not send any intelligent signals. The results showed that using AI and CFD at the same time is possible to speed up the optimization and prediction processes of membrane technology. Also, the results revealed that the costs of CFD could be reduced, and the prediction time could speed up. On the other hand, AI helped us to understand the relationship between the outputs and the inputs in the optimization process leading to have a better learning process compared to the conventional methodology. Moreover, one of the best results obtained from this study was that by increasing the number of inputs, the accuracy of prediction, and the capability of the prediction increased, and the model could predict the process in the membrane technology. Due to the low number of inputs in the training process, the model did not have the prediction capability. However, by increasing the number of inputs to 5, the regression and R value reached 0.99, which increased from 0.31 to this number. When the regression and R value reached 0.99, the capability of the model could be used for the prediction.

## List of symbols

*U*: Velocity distribution (m/s)
*P*: Pressure (Pa)
*F*: Force (N)
*w*_*i*_: Signal out-coming from the node
$\overline{{w_{i} }}$: Normalized firing strengths
*T*: Temperature (K)
*ρ*: Density (kg/m^3^)
*C*: Concentration (mol/m^3^)
*r*: Radius (m)
*η*: Dynamic viscosity (Pa s)

## Abbreviations

CFD: Computational fluid dynamics
PSO: Particle Swarm Optimization
AI: Artificial Intelligence
ANFIS: Adaptive neuro-fuzzy inference system
